# Vascular Aging: Implications for Cardiovascular Disease and Therapy

**DOI:** 10.4172/2161-1025.1000183

**Published:** 2016-08-30

**Authors:** Yohannes T Ghebre, Eduard Yakubov, Wing Tak Wong, Prasanna Krishnamurthy, Nazish Sayed, Andrew G Sikora, Mark D Bonnen

**Affiliations:** 1 Department of Radiation Oncology, Baylor College of Medicine, Houston, Texas, USA; 2 phaRNA Comprehensive RNA Technologies, Houston, Texas, USA; 3 School of Life Sciences, The Chinese University of Hong Kong, Hong Kong; 4 Department of Biomedical Engineering, University of Alabama at Birmingham, Birmingham, Alabama, USA; 5 Department of Medicine, Stanford Cardiovascular Institute, Stanford University, Stanford, CA, USA; 6 Department of Otolaryngology-Head and Neck Surgery, Baylor College of Medicine, Houston, Texas, USA

**Keywords:** Vascular aging, Endothelium, Endothelial senescence, Vascular rejuvenation, Vascular function, Vascular pharmacology, Cardiovascular health

## Abstract

The incidence and prevalence of cardiovascular disease is highest among the elderly, in part, due to deleterious effects of advancing age on the heart and blood vessels. Aging, a known cardiovascular risk factor, is progressively associated with structural and functional changes to the vasculature including hemodynamic disturbance due to increased oxidative stress, premature cellular senescence and impairments in synthesis and/or secretion of endothelium-derived vasoactive molecules. These molecular and physiological changes lead to vessel wall stiffening and thickening, as well as other vascular complications that culminate to loss of vascular tone regulation and endothelial function. Intriguingly, the vessel wall, a biochemically active structure composed of collagen, connective tissue, smooth muscle and endothelial cells, is adversely affected by processes involved in premature or normal aging. Notably, the inner most layer of the vessel wall, the endothelium, becomes senescent and dysfunctional with advancing age. As a result, its ability to release vasoactive molecules such as acetylcholine (ACh), prostacyclin (PGI2), endothelium-derived hyperpolarizing factor (EDHF), and nitric oxide (NO) is reduced and the cellular response to these molecules is also impaired. By contrast, the vascular endothelium increases its generation and release of reactive oxygen (ROS) and nitrogen (RNS) species, vasoconstrictors such as endothelin (ET) and angiotensin (AT), and endogenous inhibitors of NO synthases (NOSs) to block NO. This skews the balance of the endothelium in favor of the release of highly tissue reactive and harmful molecules that promote DNA damage, telomere erosion, senescence, as well as stiffened and hardened vessel wall that is prone to the development of hypertension, diabetes, atherosclerosis and other cardiovascular risk factors. This Review discusses the impact of advancing age on cardiovascular health, and highlights the cellular and molecular mechanisms that underlie age-associated vascular changes. In addition, the role of pharmacological interventions in preventing or delaying age-related cardiovascular disease is discussed.

## Introduction

More than three centuries ago, a famous English physician and author, Thomas Sydenham, said “A man is as old as his arteries”. This popular quote signifies a correlation between aging and the cardiovascular system including the susceptibility of this system to age-associated changes. Indeed, cardiovascular diseases such as atherosclerosis, hypertension, diabetes and heart attack are the leading causes of morbidity and mortality in the elderly population. In line with this, premature or normal aging is a major cardiovascular risk factor. According to the National Institute of Aging (NIA), about 40% of all deaths in the elderly (age 65 and older) are related to cardiovascular disease [1]. The risk for cardiovascular morbidity between the ages of 50 and 80 increases by about 10-fold. Meanwhile, premature aging syndromes such as Hutchinson-Gilford progeria syndrome (HGPS) and Werner syndrome (WRN) are disproportionally affected by cardiovascular disease including heart attack and stroke [2]. As a result, healthcare expenses related to cardiovascular care is much higher in the accelerated aging syndromes and advancing age population and has mounted profound economic and public health burden. Therefore, understanding the molecular and cell biological processes underlying age-associated structural and functional changes to the cardiovascular system including the heart and blood vessels is of significant importance.

The effect of aging on cardiovascular health is in part because aging perturbs a number of metabolic and hemodynamic mechanisms in the cardiovascular system in general and the vascular endothelium in particular [3–5]. Some of these perturbations include increased oxidative stress and reduced telomere length resulting in DNA damage, impaired replicative capacity of cells and upregulated cardiovascular tissue senescence [6]. These changes expose the heart and its vascular network to a series of risk factors that impair physiological repair mechanisms, and accelerate vascular dysfunction and cardiovascular disease.

Vascular endothelium, a diaphanous film of tissue, is the inner-most structure that coats the interior walls (tunica intima) of the cardiovascular and lymphatic systems covering a surface area of over 4,000 m^2^ [7,8]. This structure is laid with a monolayer of about one trillion endothelial cells throughout its lumen [9]. Vascular endothelial cells (ECs) have a distinct cobblestone-like morphology and are involved in the regulation of de novo formation of blood vessels (vasculogenesis), blood vessel sprouting (angiogenesis), vascular tone (vasodilation and vasoconstriction), vascular permeability, blood clotting, as well as inflammation and immune defense [8–10]. In addition, ECs are actively involved in the suppression of middle vascular layer (tunica media) cells (i.e. vascular smooth muscle cells) from outgrowing into the tunica intima layer and interfering with normal vascular function. Furthermore, ECs synthesize and release vasoactive molecules including endothelium-derived relaxing factor (EDRF) to promote relaxation of vascular smooth muscle. With advancing age, ECs have depleted anti-inflammatory and antioxidant defense mechanisms and are subjected to augmented inflammatory and oxidative stress that impairs their number, morphology and function [11]. As a result, older subjects have increased susceptibility to cardiovascular morbidity and death. The present review discusses the contribution of advancing age to vascular endothelial dysfunction, and the sequelae of aged and dysfunctional endothelium in the development and progression of cardiovascular diseases. The review also discusses current and future perspectives in the treatment of vascular aging.

### Vascular endothelium: senescence and aging

Healthy endothelium chiefly regulates cardiovascular physiology including fine tuning vascular tone, tissue perfusion and oxygenation, resistance to thrombosis, inhibition of underlying smooth muscle cell proliferation, adhesion of inflammatory cells to vessel wall and vascular fibrosis [8].

By contrast, dysfunctional or aged endothelium is characterized by several phenotypic changes and molecular patterns that include impaired replicative capacity of cells, increased cellular senescence, reduced generation of anti-inflammatory molecules, antioxidants and other salutary mechanisms that are involved in vascular homeostasis [11,12]. Physiologically, endothelial function is mediated by shear stress and the release of vasodilating molecules such as acetylcholine (ACh), prostacyclin (PGI2), adenosine, bradykinin, vascular endothelial growth factor (VEGF), nitric oxide (NO), and guanosine 3′, 5′-cyclic monophosphate (cGMP), as well as simultaneous regulation of mediators involved in vasoconstriction, inflammation and thrombosis including endothelin (ET), angiotensin (AT), prostaglandins (PGs), leukotrienes (LTs), thromboxanes (TXs), and endogenous inhibitors of NO synthases (NOSs) to block NO. Failure of the senescing endothelium to maintain the physiological balance between these opposing functions is the basis for increased cardiovascular risk in the elderly. Below is a description of how vascular aging is associated with cardiovascular morbidity and mortality.

### Is vascular senescence a risk factor for cardiovascular disease?

Cellular senescence and vascular aging are perpetrated and/or accelerated by several factors that induce oxidative stress and DNA damage. Some of the common predisposing factors include aging, tobacco smoke, chemotherapeutic drugs and exposure to radiation. For example, ionizing radiation depopulates stem cell reserve and induces premature replicative senescence (i.e. arrests cell division) of somatic cells through increased generation of free radicals and subsequent DNA damage [13–15]. In addition, some of the commonly used chemotherapeutic drugs such as doxorubicin and cisplatin induce cellular senescence by altering DNA structure and function [16]. In the cardiovascular system, some chemotherapeutic drugs and radiation therapy are associated with increased risk of cardiotoxicity and vascular damage [16–18]. In particular, the administration of radiation therapy to the chest wall is a significant cause of vascular endothelial dysfunction including impaired perfusion, fibrous thickening of the pericardium and myocardial fibrosis [19,20]. These cardiac and vascular lesions culminate to senescence of resident cardiomyocytes and endothelial cells leading to hardening of the heart muscle and stiffening of vascular wall to trigger angina, dyspnea and heart failure that leads to sudden death [21].

In the absence of history of exposure to chemotherapy or radiation therapy, traditional risk factors such as chronological aging play significant role in promoting processes involved in biological aging including oxidative stress, DNA methylation, telomere shortening, as well as structural and functional changes to the vasculature. As a result, the endothelium compromises its ability to secrete many of its vasoactive molecules such as growth factors, hormones, NO and microRNA (miRNA). In addition, the number and function of endothelial progenitor cells (EPCs) is reduced with advancing age. Taken together, the endothelium becomes senescent and prone to cardiovascular disease including hypertension, atherosclerosis and diabetes [22–24].

### Regulation of endothelial function

Exogenously, endothelial function is influenced by several factors including smoking, diet and exercise. Endogenously, the expression and/or activity of several mediators determine the integrity and optimal function of vascular endothelium including its bidirectional response to aging, disease onset and progression. Below is a description of how some key endogenous molecules, endothelial progenitor cells, as well as exercise and diet, are involved in vascular pathobiology.

#### Growth factors

A number of angiogenic growth factors and their receptors are centrally involved in the formation and maturation of blood vessels, regulation of endothelial cell proliferation and migration. Some of the main factors include vascular endothelial growth factors (VEGFs) and their receptors (VEGFR-I, VEGFR-II and VEGFR-III), platelet-derived growth factors (PDGFs) and their receptors (PDGFRalpha and PDGFRbeta), as well as fibroblast growth factors (FGFs) and their receptors (FGFR-I to FGFR-IV). By far, VEGF is the most potent and highly specific mitogen for endothelial cells. So far, five isoforms of VEGF have been identified and characterized [25,26]. The first two tyrosine-kinase receptors, VEGFR-I (Flt-1) and VEGFR-II (Flk-1/KDR), are predominantly expressed in endothelial cells and efficiently recognize most of the members of the VEGF family in order to promote cell proliferation, migration and inhibition of apoptosis. In vivo, VEGF is absolutely required in the modulation of angiogenesis and vasculogenesis and its genetic suppression in animals leads to embryonic lethality due to failure of the cardiovascular system to develop [27,28].

Similarly, targeted disruption of Flt-1 or Flk-1 causes severe defects in endothelial differentiation and formation of functional blood vessels [29,30]. By contrast, overexpression of Flt-1 may promote abnormal cell migration, cell-cell and cell-matrix interactions leading to severely defective blood vessels [29]. Thus, optimal levels of VEGF and its tyrosine kinase receptors is required for homeostasis of the vascular system. However, chronological aging is unable to maintain physiological levels of VEGF. As a result, endothelial cells isolated from aged subjects show downregulation of VEGF mRNA and protein expression and impairment in their angiogenic capacity [31]. Restoration of VEGF expression using recombinant protein promotes angiogenesis in senescent rat heart [32]. Similar to VEGF, aging has been implicated in the downregulation of PDGF receptor and impairment of cell proliferation whereas re-expression of its levels enhances cell proliferation and function [33]. The FGF signaling is also normally involved in suppression of cellular senescence and vascular dysfunction [34,35]. However, inhibition of this signaling pathway results in loss of endothelial barrier function [34].

#### Cytokines and adhesion molecules

Several cytokines can modulate vascular function directly or indirectly by regulating the expression of growth factors such as VEGF [36–39]. For example, systemically secreted or locally released tumor necrosis factor alpha (TNFα) induces macrovascular and microvascular dysfunction in part through transcriptional upregulation of pro-inflammatory cytokines including interleukins (e.g. IL6 and IL8), adhesion molecules (e.g. VCAM1, ICAM1 and MCP1), nuclear factor kB (NFkB), inducible NOS (iNOS), nicotinamide adenine dinucleotide phosphate (NADPH) oxidase, and by increasing intravascular production of ROS [40]. With age, the expression of TNFα increases, in part due to accumulation of advanced glycation end-product (AGEs), and is associated with superoxide (O^2−^) production and vascular oxidative stress [41–43]. The oxidative stress in the vessel walls is likely caused and/or exacerbated by NADPH oxidases, xanthine oxidase and uncoupled NOS [44–46]. The increase in NADPH-dependent free radical production is involved in the biotransformation of NO into tissue reactive and harmful nitrogenous species including peroxynitrite (OONO^−^). As a result of this TNFα-induced NO depletion and upregulation of pro-inflammatory gene expression, the vasculature is unable to optimally maintain its ability to relax and becomes exposed to the onset of vascular diseases including atherogenesis, thrombosis, vascular inflammation and fibrosis. By contrast, anti-TNFα treatment suppresses several markers of endothelial dysfunction including oxidative stress, pro-apoptotic markers and pro-inflammatory gene expression including iNOS. As a result, response of the endothelium to vasoactive molecules such as acetylcholine is improved [47].

#### Nitric oxide synthases

In mammalian cells, three isoforms of NOS are differentially expressed in neurons (neuronal NOS; nNOS), immune cells (inducible NOS; iNOS) and vascular cells (endothelial NOS; eNOS) [48–50]. In the cardiovascular system, eNOS is a multisubstrate enzyme and plays a significant role in catalyzing the production of NO from its substrate L-arginine in the presence of cosubstrates and cofactors [49]. Bioactive eNOS is tightly regulated and requires dimerization and phosphorylation by a protein kinase [51]. The eNOS-derived NO is multifunctional and significantly contributes to vascular homeostasis including atheroprotection, inhibition of vascular inflammation, and suppression of neointimal lesion and thickening. These protective effects of NO are important in opposing vascular diseases such as restenosis, atherosclerosis and vasculopathy. Aging of the vascular system disrupts many of these physiological processes and causes and/or accelerates eNOS uncoupling where the enzyme is involved in generating superoxide radicals and inducing vascular oxidative stress. In addition, eNOS expression and/or activity are impaired by cytokines that are pathologically upregulated with advancing age. For example, in aortic and microvascular ECs, cytokines such as TNFα and IL1β selectively downregulate eNOS expression and upregulate iNOS levels. These imbalances in NOS expression reduce bioactive NO and increase iNOS-driven OONO^−^ and ROS production to favor vascular oxidative and nitrosative stress and eventually lead to endothelial dysfunction [52–54]. By contrast, inhibition of iNOS expression in aged animals reduced nitrosative stress and reversed endothelial dysfunction [55].

Other age-associated factors that influence NOS expression and/or activity to quench NO levels include: i) reduction in the levels of NOS cosubstrates and cofactors including flavin adenine dinucleotide (FAD), Flavin mononucleotide (FMN) and tetrahydrobiopterin (BH4) [49]; ii) increased arginase activity and alternate metabolism of L-arginine [56]; iii) replicative senescence of vascular cells including endothelial and smooth muscle cells [57–59]; and iv) elevation of asymmetric dimethylarginine (ADMA) levels [60,61]. ADMA is a methylated arginine that endogenously and competitively inhibits eNOS to reduce bioavailability of NO and accelerate endothelial cell senescence [62]. Plasma level of ADMA is an independent predictor of major adverse cardiovascular events [63]. Physiologically, ADMA is metabolized by dimethylarginine dimethylaminohydrolase (DDAH) [64]. However, DDAH expression and/or activity is impaired by oxidative stress including oxidized lipids and TNFα [64,65]. Thus, each of these factors could lead to increased vascular stiffness, reduced endothelium-dependent vasodilation and accelerated vascular dysfunction.

#### MicroRNAs

MicroRNAs (miRNAs), single-stranded RNAs of about 25 nucleotides that endogenously suppress protein expression by destabilizing target mRNAs, play profound role in the pathobiology of the cardiovascular system. Several gain- and loss- of function preclinical studies have shown that miRNAs are useful in understanding molecular mechanisms of cardiovascular disease and as novel drug targets to treat cardiac and vascular pathologies. In addition, pharmacologic manipulation of miRNAs using oligonucleotides have shown some promise in attenuating cardiovascular disease and in defining a path for miRNA-based therapeutics. Mechanistically, miRNAs interact with Argonaute (AGO) protein that is complexed with RNA-induced silencing complex (RISC) proteins in the cytosol of cells in order to recognize and target the 3′ untranslated regions (UTRs) of mRNA transcripts and inhibit their protein translation; several targets at a time. In order to mount their sustained effect in the cardiovascular system, miRNAs are loaded into vesicles, lipid particles and exosomes before being released into the bloodstream. Of the nearly 1000 miRNAs in the human genome, several are implicated in the pathogenesis of heart and vascular disorders including angiogenesis, vessel growth, inflammation and fibrosis. Some of the well-characterized vascular miRNAs include miR-17-92 cluster, miR-29, miR-30, miR-31, miR-34a, miR-43a, miR-126, miR-143, miR-145, miR-146 family, miR-216, miR-217 and miR-299 [66–68]. Each of these miRNAs target critical cellular processes that are involved in senescence.

During endothelial senescence and dysfunction, the family members of miR-29 and miR-34a are significantly upregulated. For example, the miR-29 family of miRNAs is overexpressed in senescent ECs and cardiovascular tissue [69,70] likely due to DNA damage-mediated dysregulation of tumor suppressor mechanisms and cell senescence mediators [71,72]. In a senescence mouse model of Klotho-deficiency, induction of miR-29 expression was associated with decreased expression of matrix proteins and accelerated tissue aging [70]. Similarly, miR-34a overexpression inhibits EC proliferation and induces senescence by suppressing the expression of sirtuin 1 (SIRT1); a histone deacetylase that controls various cellular processes including cellular plasticity and senescence [73]. In addition, the expression of miR-34a is upregulated in tissues explanted from older animals [73–76].

#### Sex steroid hormones

Chronological and vascular aging are known to be distinctively regulated in men and women. This gender-based difference in vascular (dys)function culminates to differences in cardiovascular risk [77,78]. Sex hormones in general, and estrogen and testosterone in particular are primarily implicated in these pathophysiological differences. Several clinical studies indicate that estrogen plays a protective role in women during their child-bearing age. As a result, the prevalence of heart disease is differentially higher in men than women until at least menopause and advancing age dampen endogenous estrogen production and negate the differences [78]. In this regard, other studies also report that estrogen has vasoprotective function in premenopausal women [79–81]. As part of this protective mechanism, it has been demonstrated that estrogen increases the expression and activity of eNOS [82], reduces ADMA levels [83] and scavenges free radicals [84]. This modulation of endothelium-derived vasoactive molecules together with the action of estrogen on lipid metabolism may be the mechanism of action for estrogen’s cardioprotective effect.

In a mechanistic study to understand the vascular effect of estrogen pre- and post-menopause, Novella et al. [85] exposed uterine arteries from postmenopausal women to vehicle, synthetic estrogen or raloxifene (anti-estrogenic molecule on the uterus). The data from this study shows that estrogen reduced the levels of several classic proinflammatory markers including TNFα and IL1β. Interestingly, this study revealed biphasic effect of estrogen as a function of aging where 5 years postmenopausal age was associated with a reversed function of estrogen from anti-inflammatory to a pro-inflammatory modulator of vascular inflammation. This switched effect of estrogen has been linked to increased expression of estrogen receptors with aging [85]. This phenomenon likely explains the mixed effect of estrogen replacement therapy in aged postmenopausal women [86,87].

Meanwhile, some studies have reported that testosterone induces endothelium-independent relaxation of vascular beds [88,89] and endothelial release of NO through extracellular signal-regulated kinase (ERK)/mitogen-associated protein kinase (MAPK) pathway [90]. The later effect is likely mediated by the endogenous conversion of testosterone into estrogen [91]. With aging, testosterone levels decrease by about 2% per year and this sustained reduction may contribute to vascular aging. This possibility is supported by preclinical data that found resistance of vessels isolated from aged animals to testosterone-mediated vasodilation compared to blood vessels from younger animals [92] and by clinical data that demonstrate regulation of vascular function by testosterone replacement therapy [93,94].

#### Endothelial progenitor cells

Endothelial progenitor cells (EPCs) are presumed as the precursor cells for mature ECs and are, albeit controversially, reported to be involved in the repair and regeneration of denuded endothelium including in neovascularization [95,96]. EPCs, characterized by the expression of a set of molecular markers including CD34, CD133 and KDR, are normally isolated from bone marrow and blood (peripheral or umbilical cord). In vitro, they can be differentiated into mature CD31-expressing ECs in the presence of appropriate growth factors including VEGF, FGF and EGF [97]. In vivo, EPCs secrete angiogenic factors such as VEGF and HGF, migrate and adhere to injured vessels to promote revascularization and repair of ischemic tissue [98]. Several studies report that the number and function of EPCs including their proliferative and migratory capacity is reduced with age in part due to activation of pro-senescent and pro-oxidant pathways [99]. In addition, cardiovascular risk factors such as diabetes, atherosclerosis and hypertension also accelerate premature senescence of EPCs and impair the function of mature ECs [100]. As a result, injury to the endothelium fails to physiologically repair in older subjects or patients with cardiovascular disease.

In a mouse model of hindlimb ischemia, transplantation of bone marrow-derived EPC-like cells isolated from patients with chronic ischemic cardiomyopathy showed poor function in restoring tissue perfusion compared to the same number of cells isolated from normal subjects [101]. Clinically, transplantation of EPCs in patients with peripheral arterial disease (PAD) have been demonstrated to increase the number of collateral vessels, and improve blood flow [102].

Overall, based on preclinical and early phase clinical studies, it appears that EPCs actively participate in vascular homeostasis, and their function is significantly impaired by age and other cardiovascular risk factors in part due to suppression of angiogenic cytokines and growth factors, as well as co-influence of age and cardiovascular disease on EPC senescence including telomere length and antioxidant defense mechanisms. In this regard, several studies demonstrated that there is increased loss of telomere length and/or upregulated expression of senescence markers in EPCs treated with pro-oxidant molecules such as high glucose, angiotensin II and oxidized LDL [102]. By contrast, treatment with antioxidants or forced expression of telomerase increases EPC proliferation and vasculogenesis [103,104].

#### Progeroid syndromes and the endothelium

Premature aging syndromes such as Fanconi anemia, dyskeratosis congenita (DKC), Hutchinson-Gilford progeria syndrome (HGPS), Bloom syndrome (BS), Cockayne syndrome (CS) and Werner syndrome (WRN) are characterized by accelerated aging phenotype that affects several mesodermal tissue types including the vascular endothelium. One common feature of all these syndromes is that they result from genetic mutations of corresponding genes encoding DNA repair machinery or translation of lamin A protein (progerin); as in HGPS [105]. In HGPS, cardiovascular disease is the leading cause of death in part due to derangement of vascular tissue including decellularization of the intimal and medial layer and calcification of the vessel wall [106,107]. In this regard, the transgenic mouse model created by Francis Collins’ group at the NIH [107] provided significant insights into understanding the vascular pathobiology of human progerin overexpression. Structurally, examination of blood vessels revealed that the medial layer became thick and lost its resident vascular smooth muscle cells. In addition, the explanted arteries were found to be calcified and fibrosed [107]. Functionally, the blood vessels lacked response to the vasoactive molecule sodium nitroprusside.

Mechanistically, HGPS is caused by accumulation of truncated lamin A protein that has a farnesyl modification [108–110]. The truncation and uncleaved farnesyl moiety impedes the protein from being released out of the nuclear membrane causing destabilization of the nuclei, DNA damage, oxidative stress and telomere erosion [111]. Recently, pharmacological agents that block the farnesylation of lamin A precursor (prelamin A) and its truncated version progerin have been developed and shown to reduce many of the cardiovascular features seen in progeroid children [112–114].

#### Diet, exercise and vascular health

The pathobiologic effect of metabolic diseases such as atherosclerosis and diabetes on the cardiovascular system in general, and the endothelium in particular is well characterized [115]. As such, hypercholesterolemia and hyperglycemia have long been known to induce oxidative stress, promote vascular inflammation, and increase the risk of coronary arterial disease [115,116]. For example, high-fat diet has been shown to impair vascular function, and dyslipidemic patients have more severe endothelial dysfunction than healthy controls with normal baseline plasma triglyceride profile [117]. Dysfunctional endothelium increases the risk of developing insulin resistance, which further augments the risk for hypertension, type 2 diabetes and other metabolic syndromes [116,118].

In diabetics, high-fat and/or carbohydrate-enriched diet increase circulating levels of several pro-inflammatory molecules including iNOS, TNFα, interleukins and adhesion molecules; chemicals that are known to potentiate processes involved in endothelial dysfunction including vascular inflammation and senescence [119]. By contrast, inhibition of these pro-inflammatory cytokines using antibodies or small molecules have been shown to reduce adhesion of inflammatory cells into ECs and restore endothelial responsiveness to vasoactive molecules [47,120].

While high fat/carbohydrate diet may accelerate endothelial dysfunction, epidemiological studies have mounted evidence that traditional Mediterranean diets that limit saturated fats, and enrich fruits and leafy green vegetables reduce endothelial dysfunction and cardiovascular risk [121].

In addition to plant-based diets and pharmacological therapy, regular physical exercise (45 min to 1 hour per day to burn about 300 Kcal of energy) has been shown to reduce levels of circulating proinflammatory molecules and favor sustained release of endogenous factors that are associated with good endothelial health including anti-inflammatory molecules, antioxidants, hormones and vasodilators such as NO, prostacyclin and adenosine. Among the mechanisms by which physical exercise improves vascular health are induction of laminar shear stress and improved blood flow [122–124]. These are endothelial-dependent mechanisms that significantly affect vascular structure and function including favorable changes in vessel diameter, as well as endothelial NOS expression and activity [125].

Regular physical exercise has therefore been linked to lean body mass, normal blood pressure, improved plasma sugar, insulin and lipid profiles [126–128]. Accordingly, physically active individuals have relatively reduced loss of vascular function and lower risk of cardiovascular morbidity and mortality [129,130].

### Pharmacological regulation of vascular senescence and aging

Several molecular and cell biological studies have unraveled genes and pathways that drive and/or accelerate vascular senescence [131,132]. The studies have also identified druggable targets to improve vascular compliance including endothelium-dependent vasodilation, and thereby reduce cardiovascular morbidity. By extension, preclinical and clinical studies have tested the efficacy of several FDA-approved drugs and new chemical entities (NCEs) to modify chronological aging- or premature aging-associated vascular dysfunction.

Among the mainstream targets of currently pursued drug therapy are the NOS pathway, cyclooxygenase (COX) pathway, the renin-angiotensin system (RAS), pro-inflammatory pathways and telomerase [40,131,133]. One common goal of these therapeutic modalities is to preserve or improve vascular function and structure by suppressing interdependent processes including oxidative stress, inflammation and telomere attrition. For example, HMG-CoA reductase inhibitors (e.g. statins) have been shown to prevent senescence and improve proliferative capacity of EPCs through modulation of cell cycle genes, pro-oxidants, and pro-inflammatory pathways [134,135]. Similarly, the COX-2 inhibitor aspirin has been reported to attenuate replicative senescence of ECs through inhibition of oxidative stress, as well as induction of NO. Interestingly, the effect of aspirin on NO was found to be due to modulation of the endogenous NOS inhibitor ADMA [136]. Another study has demonstrated that ADMA induces vascular senescence by accelerating telomere shortening and reducing telomerase activity in ECs [62].

In addition to HMG-CoA reductase and COX inhibitors, inhibitors of RAS have shown some beneficial effect on the vascular system. Treatment of vascular cells with angiotensin II (Ang II) accelerates senescence and its pharmacological inhibition with olmesartan prevents the onset of Ang II-induced vascular inflammation and premature senescence [137]. Clinical studies also showed that treatment with valsartan (Ang II inhibitor) or quinapril (ACE inhibitor) improved vascular compliance in elderly subjects [133,138]. Among the pleiotropic mechanisms of statins and ACE inhibitors in modulating vascular inflammation is the inhibition of circulating TNFα levels [139,140]. In this regard, several TNFα inhibitors including etanercept and infliximab have been demonstrated to inhibit intravascular TNFα and reverse endothelial dysfunction [141,142].

In principle, another intriguing target to slow or reverse vascular aging is the telomerase enzyme complex including telomerase reverse transcriptase (TERT), telomerase RNA (TERC), dyskerin, and subunits of the shelterin complex. This conviction stems from the common occurrence of telomere shortening with advancing age or in premature aging syndromes [143]. Short telomeres increase the likelihood of DNA damage, genomic instability and mutagenesis. In addition, almost every post-mitotic cell type, with the exception of germline cells, express negligible levels of telomerase rendering them to be exquisitely susceptible to telomere-dependent replicative senescence; a phenotype that enhances the generation of ROS and pro-inflammatory molecules in vascular cells [58,144]. By contrast, endothelium-derived NO delays the onset of EC senescence through TERT activation [145]. Proof-of-concept genetic studies have reported that TERT reactivation increases longevity and favorably modulates cardiovascular disease including insulin sensitivity [146]. Similarly, activation of telomerase using phytochemicals such as resveratrol has been reported to delay EPC senescence and improve their proliferative and migratory capacity [147].

**Figure 1:**
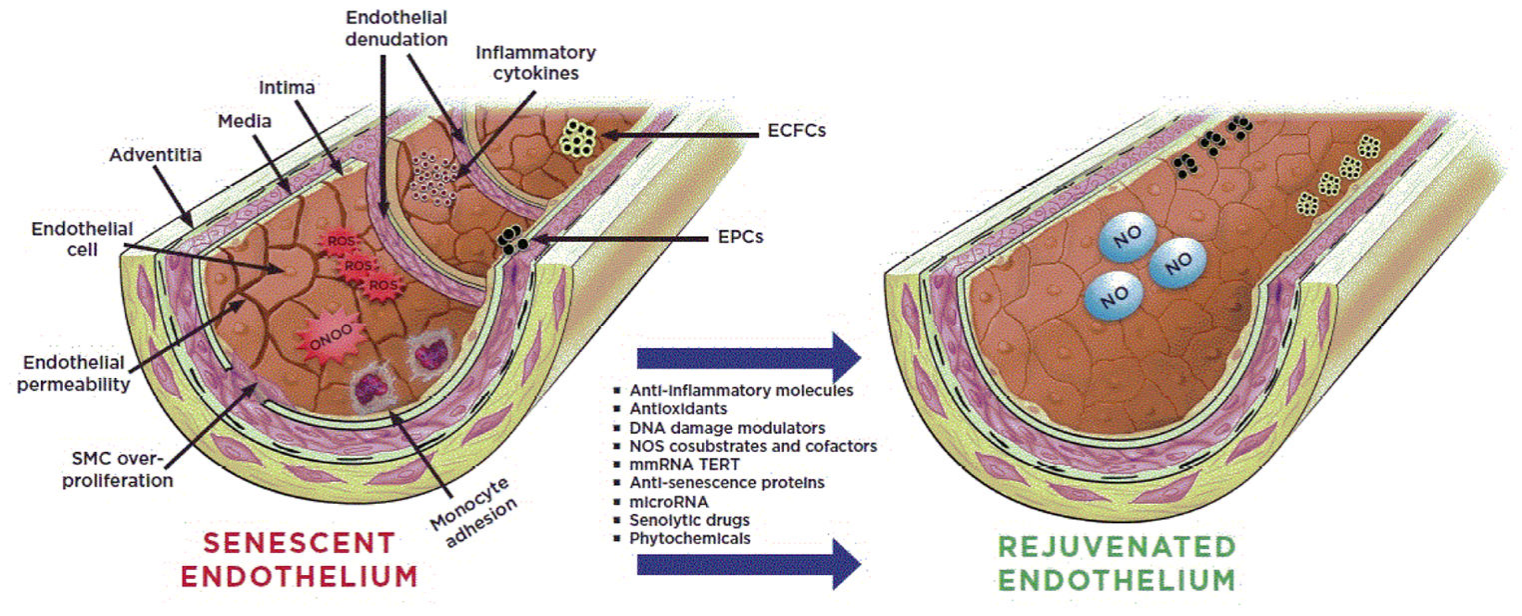
Senescence and rejuvenation of vascular endothelium. A) Aging or vascular injury contributes to endothelial dysfunction and senescence including increased endothelial permeability, over-proliferation of smooth muscle cells (SMCs), adhesion of inflammatory monocytes, accumulation of reactive oxygen species (ROS), peroxynitrite (OONO^−^), and inflammatory cytokines. In addition, the number of endothelial precursor cell reserve such as endothelial progenitor cells (EPCs) and endothelial colony-forming cells (ECFCs) is markedly reduced. In B) a rejuvenated vascular endothelium, as a result of pharmacological intervention, characterized by continuous monolayer of endothelial cells and production of beneficial vasoactive molecules such as nitric oxide (NO), as well as enriched EPCs and ECFCs is shown.

More recently, it has been demonstrated that introduction of exogenously expressed TERT in the form of modified messenger RNA (mmRNA) increases TERT enzymatic activity and transiently extends telomere length in primary human cells [148]. This technology may be able to delay cellular and tissue senescence, and increase the proliferative capacity of cells without immortal transformation. Systemic and transient delivery of such mmRNA formulated with suitable carriers to senescent endothelium is expected to slow or even reverse vascular aging and dysfunction (Figure 1).

## Conclusion and Perspectives

Currently, considerable efforts are being made to delay aging and age-associated diseases including cardiovascular morbidity. Endothelial dysfunction is one of the earliest indicators of cardiovascular disease. In line with this, the endothelium has emerged as one of the most important targets for the prevention and treatment of cardiovascular disease including hypertension, diabetes, atherosclerosis, and insulin resistance syndrome. ECs, mature or progenitor, are the building blocks of the vascular endothelium and are involved in active secretion of paracrine factors to modulate vascular homeostasis. Unfortunately, aging exerts several pathological changes in the vascular system including apoptosis, inflammation, DNA damage and telomere attrition. As a result, ECs lose their ability to proliferate and secrete vasoactive molecules. Several of the existing strategies attempt to restore key EC functions including production of NO (through exogenous supplementation or reactivation of co-substrates and cofactors) and other vasodilators while decreasing inflammation, oxidative and nitrosative stress through anti-inflammatory, antioxidants and restoration of eNOS coupling. However, these strategies have not been able to rejuvenate denuded or senescent endothelium in a meaningfully way.

In order to effectively overcome the exhausted number and function of mature ECs, endothelial lineage progenitors such as EPCs and endothelial colony-forming cells (ECFCs) [149,150] may be isolated from circulation or from niches within the vascular wall and rejuvenated through ectopic expression of factors that halt senescence and other age-associated phenotypes. In this regard, transient extension of telomere length through non-viral and non-integrating approaches ex vivo is particularly appealing. This cell-based strategy may be combined with other mechanisms involved in the regulation of cellular senescence such as microRNA, senolytic drugs and/or NCEs that modulate DNA damage repair for preventative or therapeutic vascular rejuvenation (Figure 1).
